# A Facile Molding Method of Continuous Fiber-Reinforced Thermoplastic Composites and Its Mechanical Property

**DOI:** 10.3390/polym14050947

**Published:** 2022-02-26

**Authors:** Jian Shi, Mamoru Mizuno, Limin Bao, Chunhong Zhu

**Affiliations:** 1 Faculty of Systems Science and Technology, Akita Prefectural University, Akita 015-0055, Japan; mizuno@akita-pu.ac.jp; 2 Faculty of Textile Science and Technology, Shinshu University, Nagano 386-8567, Japan; baolimin@shinshu-u.ac.jp; 3 Interdisciplinary Cluster for Cutting Edge Research (ICCER), Institute for Fiber Engineering (IFES), Shinshu University, Nagano 386-8567, Japan

**Keywords:** C-FRTP, molding method, pre-impregnated materials, mechanical property, high fiber volume fractions

## Abstract

The mechanical properties of continuous fiber-reinforced thermoplastic (C-FRTP) composites are commonly lower than those of continuous fiber-reinforced thermosetting plastic (C-FRP) composites. We have developed a new molding method for C-FRTP. In this study, pre-impregnated materials were successfully prepared by polymer solution impregnation method and, finally, C-FRTP was fabricated. The viscosity of the thermoplastic matrix was decreased to approximately 3dPa×s, the same level of epoxy, and the fiber volume fraction was increased from approximately 45 to 60%. The cross-section of specimens were polished by an ion milling system and impregnation condition was investigated by scanning electron microscopy (SEM). The micrographs suggested that thermoplastic polymer was impregnated to every corner of the fiber, and no void was found on the cross-section. It revealed that void-free composites with perfect mechanical properties can be manufactured with this new molding method. All specimens were submitted to a mechanical measuring equipment, and the mechanical properties of the composite specimens were investigated. Mechanical analysis revealed that tensile property and flexural property of C-FRTP were enhanced up to the same level with C-FRP.

## 1. Introduction

Fiber-reinforced plastic (FRP), a composite material made of a polymer matrix reinforced with fibers, has been widely used in various industries for their high specific strength and stiffness, such as automobiles, aeroplanes, watercrafts, windmill blades, tennis rackets and so on. The reinforced fiber in FRP can be present in approximately three formats, e.g., short fiber, long fiber, and continuous fiber, and classified according to its shape, aspect ratio, geometric arrangement and concentration. FRP, with continuous fiber-reinforcement, offers significant improvement in stiffness and strength compared to that with short fiber or long fiber [1,2,3,4]. The polymer matrix of FRP can be classified as either thermosetting matrix or thermoplastic matrix. FRP with thermoplastic matrix, commonly called FRTP, offers higher fracture toughness, higher impact resistance, shorter processing cycle times, better recyclability, re-formability, and repairability compared to that with thermosetting matrix [5,6,7]. 

However, it is very difficult to fabricate continuous fiber-reinforced thermoplastic with the current major technologies, e.g., injection molding which was developed for manufacturing short fiber-reinforced thermoplastic (S-FRTP), and vacuum assistant resin transfer molding (VaRTM), for C-FRP. The reason could be attributed to high viscosity of thermoplastic matrix. During the impregnation process, viscosity affects impregnability of fibers, which is critical for mechanical properties of FRP composites. High viscosity leads to low mechanical properties because it is hard for high viscosity polymer matrix to impregnate fiber completely, with abundant bubbles and polymer-matrix-free regions forming in final FRP composites. These can lead to stress concentrations resulting in poor mechanical properties.

In order to fabricate C-FRTP with excellent mechanical properties, a new molding method should be developed not only for FRP composites production, but also for a wider range of composites material manufacturing; for example, serving as a new basic method for producing thermoplastic biocomposite [8,9] and other functional composite materials [10,11,12]. Currently, there are two main types of technologies being used for FRTP [13,14,15,16]. One is as close to the fiber and thermoplastic polymer matrix as possible before the final step in composite fabrication, such as powder impregnation, commingled yarn, co-woven fabric, film-stacking method technologies, to reduce void; the other is to synthesise the thermoplastic polymer matrix directly, such as in situ polymerization, to reduce viscosity [5]. Powder impregnation technique typically continuously pulls the reinforcing fiber yarn through powdered thermoplastic resin beds, pins or rollers, usually being used to open up the fiber tow and allow the thermoplastic powder fall into fiber gaps [5,17,18]. Commingled yarn technologies is to commingle the reinforcing fibers and thermoplastic fibers in a single yarn, to minimize the distance between reinforcing fibers and thermoplastic resin for impregnation [19,20]. Recently, commingled yarn technology is combined with 3D printing technique for production on C-FRTP [21]. Co-woven fabrics are woven through reinforcing fiber yarns and thermoplastic fiber yarns, and they can easily fabricate 3D composites as raw materials compared with current technologies [22,23]. Of course, the distance between reinforcing fibers and thermoplastic resin is much longer than in the case of commingled yarn. In the film-stacking method, without going through prepreg, fiber sheets are used directly. They are laminated alternately with thermoplastic film, then heated and pressed. When the temperature rises above the melting point of the thermoplastic polymer, the film melts and penetrates into the fiber sheet. When the temperature cools down to room temperature, it is solidified and molded [24,25,26]. An in-situ polymerization method was developed recently, whereby the reinforcing fibers are impregnated by monomer with low viscosity, which is a chemical substance before it becomes a thermoplastic polymer. FRTP can obtained when polymerization reaction occurs in situ [27,28,29,30]. Most methods mentioned above focused on fiber bundle level, and difficult to achieve without an especial equipment. 

Since the above methods all require preprocessing of thermoplastic resins into powders, fibers, films, or require a high degree of synthesis technology, it is difficult to promote in practical applications. In order to achieve simpler manufacturing of composite materials, we have developed a new composite material fabrication method. The present work reports that a facile method was developed to achieve low viscosity thermoplastic matrix so that the impregnation was improved, and fiber volume fraction was increased in final C-FRTP composites [31]. A convenient process of producing pre-impregnated material by polymer solution impregnation method is proposed. 

## 2. Materials and Methods

### 2.1. Materials

The matrix material used in this work was a commercial thermoplastic polyester resin Vylon (Toyobo Co., Ltd., Osaka, Japan) and thermosetting epoxy resin (XNR/H 6815, Nagase ChemteX Corporation, Osaka, Japan). The physical properties of these matrix resin are presented in Table 1. The reinforcing fiber used in this work was a commercially available plain woven fabric glass fibers (WF230100BS6, Nitto Boseki Co., Ltd., Tokyo, Japan) and PAN-based T300 plain woven fabric carbon fibers (CO6343, Toray Industries Inc., Tokyo, Japan). N-Methyl-2-pyrrolidone (NMP, FUJIFILM Wako Pure Chemical Industries, Ltd., Osaka, Japan) was used as a solvent. The physical properties of these reinforcing fibers are presented in Table 2.

### 2.2. Preparation of Pre-Impregnated Sheet

Firstly, polyester resin pellets were dissolved in NMP using a ceramic hotplate magnetic stirrer (CHPS-250DH, AS ONE Corporation, Osaka, Japan) at different weight percentages (10, 15, 20, 25, 30 wt%) at 150 °C for 3 h to lower the viscosity of the thermoplastic polymer impregnating solution for better impregnation. Brookfield viscometer (DV-I Prime, Brookfield Engineering Inc., Middleboro, MA, USA) was employed to measure the rotational viscosity of the polyester resin solution and epoxy resin. The rotational speed was set as 30 r min^−1^ at 25 °C in according to JIS K 7117-1. 

**Figure 1:**
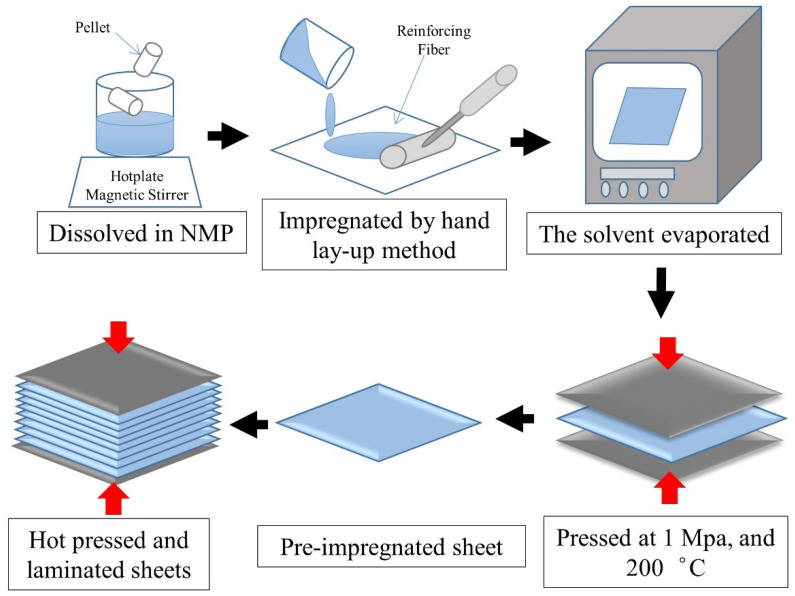
The schematic of preparation of thermoplastic matrix pre-impregnated sheet.

Pre-impregnated sheets were fabricated from continuous reinforcing fiber sheets and prepared by a polyester impregnating solution by hand in order to achieve sufficient impregnation and uniform distribution of the impregnating solution in the fiber sheet. Then, the reinforcing fibers sheet impregnated with polyester impregnating solution was removed into a vacuum oven (AVO-200V, AS ONE Corporation, Osaka, Japan) and heated at 220 °C for 6 h, which evaporated the solvent adequately. Next, the pre-impregnated sheet was placed in a hot press machine (AH-2003; AS ONE Corporation, Osaka, Japan) at 1 MPa pressure and 200 °C for 10 min to expel excess impregnating solution. Pressure of 1MPa was kept till the temperature cooled down to room temperature, then the pre-impregnated sheet was taken out from hot press machine and cut into 100 × 100 mm^2^ according to the size of metallic mold. The schematic of preparation of thermoplastic matrix pre-impregnated sheet is shown in Figure 1.

### 2.3. Preparation of C-FRTP Composite

Eight cut pre-impregnated sheets were laminated in the metallic mold, and placed in the hot press machine at 7.9 MPa for 30 min at temperature increasing from room temperature to 200 °C. After turning off the heat, pressure of 7.9 MPa was kept till the temperature cooled down to room temperature, then the specimen was taken out, which was labelled as C-GFRTP and C-CFRTP according to used reinforcing fiber. C-GFRP and C-CFRP which using thermosetting epoxy resin were molded by vacuum-assisted resin transfer molding (VaRTM) method. The fiber volume fraction was calculated according to Equation (1):

(1) $$V_{f}=\frac{M_{f}}{V_{c}\times \rho_{f}}\times 100\%$$

where *V*_f_ represents the fiber volume fraction, *M*_f_ and *ρ*_f_ represent mass of the fibers and density of the fiber, respectively. *V*_c_ represents volume of the prepared C-FRTP composite.

### 2.4. Cross-Section Polishing by Ion Milling 

**Figure 2:**
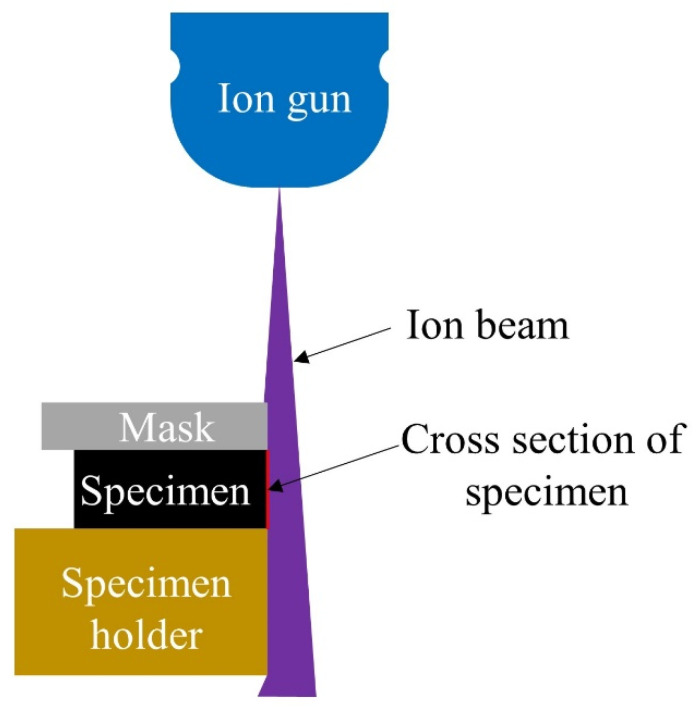
Diagram of cross section polishing by ion milling system.

In order to confirm the impregnation of the thermoplastic polyester resin, C-FRTP composite specimens were cut off, and the cross section of the specimen was polished using an ion mill system (IM 4000 Plus; Hitachi High-Tech Corporation, Tokyo, Japan). The diagram is shown in Figure 2. Then, they were submitted to scanning electron microscopy (SEM) for detailed observation.

### 2.5. Mechanical Testing

The mechanical properties of all specimens were tested using a universal testing machine (EZ-Graph, Shimadzu, Kyoto, Japan). According to JIS K 7164, tensile test was carried out with a crosshead speed of 1 mm/min to determine the tensile strength and modulus of all specimens. A strain gauge was used for accurate determination of the tensile modulus. Three point flexural test was conducted with a crosshead speed of 1 mm/min according to JIS K 7017. The value of 10 tests was averaged.

### 2.6. Microscopy Analysis

SEM (S-3000N, Hitachi High-Tech Corporation, Tokyo, Japan) was employed to determine impregnation of the thermoplastic polyester resin, and fractured morphology. All specimens were coated with a thin layer of gold before observation. SEM images were obtained at an acceleration voltage of 10 kV under high vacuum mode.

## 3. Results and Discussion

### 3.1. Investigation of Matrix Resin Viscosity

**Figure 3:**
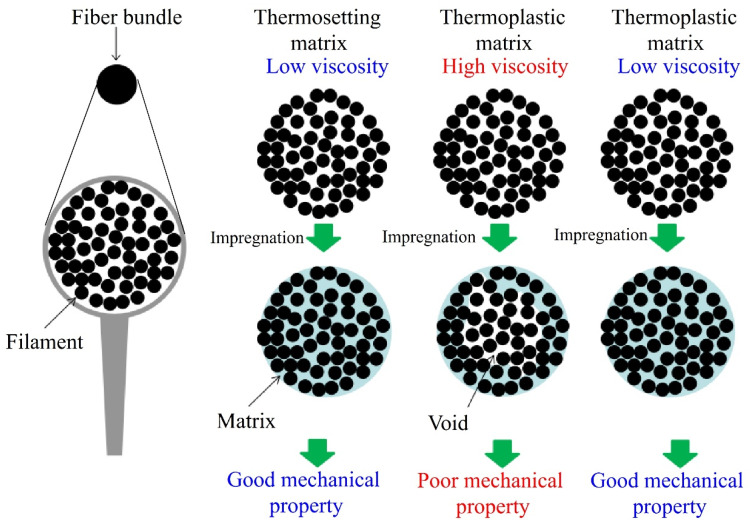
Diagram of relationship between viscosity and impregnation.

**Figure 4:**
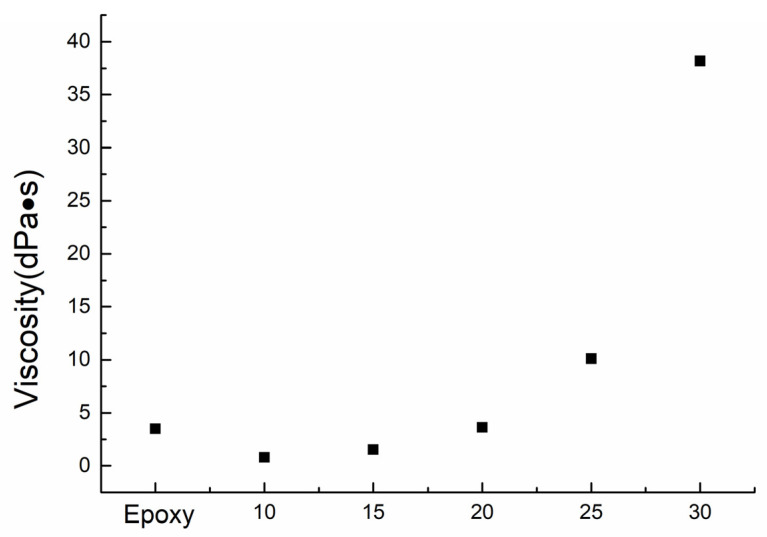
Viscosity of epoxy resin and polyester resin solution at different mass fraction.

Viscosity is of the essence in the impregnation process of C-FRTP molding. A single fiber is so thin that the diameter is only approximately 10 μm. It is conceivable that the space between fibers is very narrow, limiting the high viscosity matrix resin flow into the space, which causes a great deal of space to be unfilled with matrix resin, leading to low mechanical properties. Figure 3 shows the diagram of relationship between viscosity and impregnation. Therefore, the present work tried to reduce the viscosity of thermoplastic matrix resin for improving impregnation using solvent solving method. Figure 4 shows the viscosity of thermosetting epoxy resin and thermoplastic polyester resin solution at different mass fraction. The viscosity value of thermosetting epoxy was 3.48 dPa×s. The viscosity values of the different mass fraction thermoplastic polyester resin solutions were 0.79, 1.53, 3.62, 10.09, and 38.17 dPa×s, respectively. Generally, this shows an increase in viscosity with increasing mass fraction. As shown in Figure 4, the viscosity values of 10 wt%, 15 wt% and 20 wt% of polyester resin solution were the same level with epoxy resin, which leads to perfect impregnation. In fact, the viscosity values were lower than that of epoxy resin under the condition of 10 wt% and 15 wt%, which indicated that the impregnation was easier than epoxy resin. However, under the conditions of 10 wt% and 15 wt%, the amount of matrix resin was not enough, so it lead to voids between fibers and caused low mechanical properties. The viscosity values of 25 wt% and 30 wt% were higher than that of 10 wt%, 15 wt% and 20 wt% and epoxy resin. In particular, when the mass fraction was 30 wt%, the viscosity value was almost 10 times that of the epoxy resin. High viscosity of the thermoplastic polyester resin makes infusion difficult, resulting in poor impregnation into fibers, ultimately leading to insufficient interfacial adhesion during the composite fabrication process and low mechanical properties. Therefore, the mass fraction of polyester resin used in this work is 20 wt%, of which the viscosity value is almost the same as the epoxy resin. Therefore, this is a facile method to achieve low viscosity matrix resin. It can be confirmed that the viscosity values are decreased effectively when compared to the melting viscosity of polyester resin, which is approximately 7000 dPa×s at 250 °C.

### 3.2. Fiber Volume Fraction and Mechanical Property

**Figure 5:**
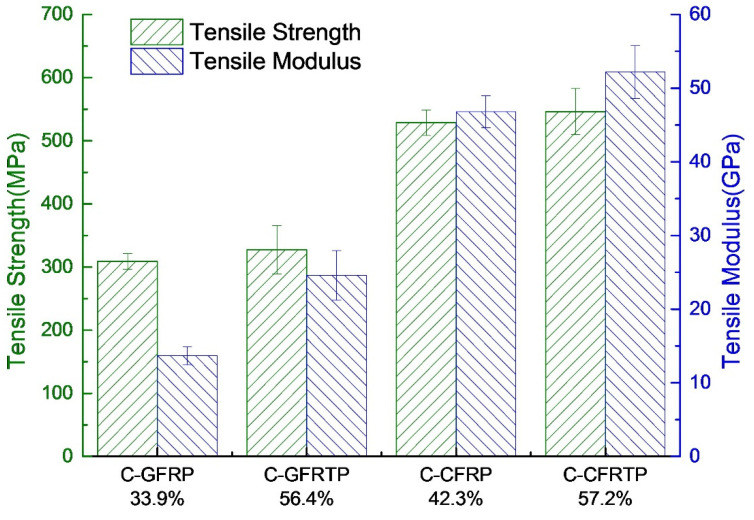
Comparison of tensile property of C-GFRP, C-GFRTP, C-CFRP and C-CFRTP.

**Figure 6:**
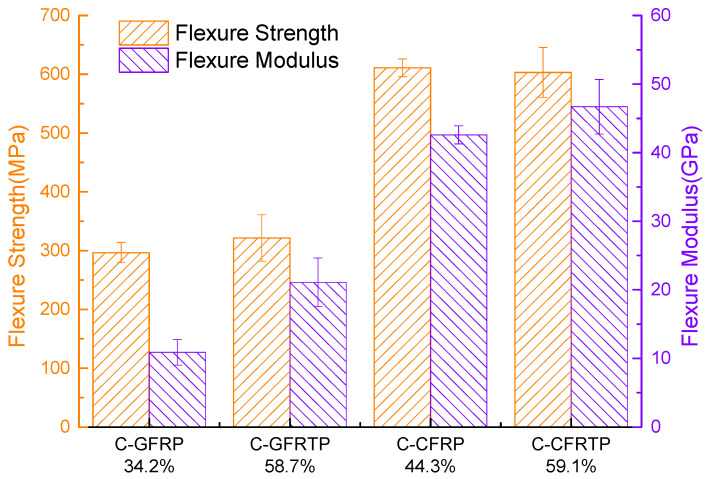
Comparison of flexural property of C-GFRP, C-GFRTP, C-CFRP, C-CFRTP.

The fiber volume fractions of all specimens are listed in Table 3. The fiber volume fraction of C-FRTP was increased to approximately 60% by the present work compared to the approximately 40% of the C-FRP fabricated by the VaRTM method [32]. Furthermore, the tensile property and flexural property are summarized in Figure 5 and Figure 6, respectively.

Figure 5 demonstrates the effect of the type of matrix resin on the tensile property of the C-FRP composite. C-GFRP and C-CFRP were fabricated from continuous glass fiber/epoxy resin and continuous carbon fiber/epoxy resin by the VaRTM method, respectively. C-GFRTP and C-CFRTP were fabricated from continuous glass fiber/polyester resin and continuous carbon fiber/polyester resin by method developed by the present work, respectively. Green pillars show the tensile strength of C-GFRP, C-GFRTP, C-CFRP and C-CFRTP, while blue pillars show the tensile modulus of these specimens. The fiber volume fractions show at the button of Figure 5. C-GFRP and C-GFRTP, C-CFRP and C-CFRTP show similar tensile strength. The tensile modulus of C-GFRP and C-CFRP are a little lower than that of C-GFRTP and C-CFRTP. Generally, the mechanical properties of FRP are better than that of FRTP. Since the viscosity of thermosetting resin before molding is much lower than that of thermoplastic resin, it can fully impregnate between single fibers to reduce the void and improve mechanical properties. Therefore, the tensile strength of both C-GFRTP and C-CFRTP produced by the method of the present study reaches the same level as that of C-GFRP and C-CFRP formed by thermosetting resin, which is an unexpected and very satisfactory result. The fiber volume fraction, the mechanical properties of reinforcing fibers and matrix resin, and the interfacial property between reinforcing fiber and matrix resin will influence the tensile property of FRP composites [33,34]. It can be observed from Figure 5 that although the tensile strength of all specimens, which were fabricated from same method and fibers, are similar, the fiber volume fractions are different. On the premise of the same fiber volume fraction, the tensile strength of C-GFRP and C-CFRP is much higher than that of C-GFRTP and C-CFRTP. In other words, the tensile strength of the FRP fabricated from epoxy is higher than that fabricated from polyester resin. This is attributed to the lack of interfacial bonding between reinforcing fiber and polyester resin. It can be considered that the surface sizing agent used to treat the fibers before they left the factory was not suitable for use with polyester resin. Fiber could not transfer the load to matrix resin well. The interfacial bonding between reinforcing fibers and epoxy resin is stronger than that between the fibers and the polyester resin [34]. The tensile modulus of C-GFRTP and C-CFRTP are higher than C-GFRP and C-CFRP. This should be attributed to the high fiber volume fraction. Since the tensile modulus of the reinforcing fiber is significantly higher than that of the resin (generally 10–100 times higher than that of the resin), the results should be related to the high fiber content of C-GFRTP and C-CFRTP. The tensile strength of C-GFRTP fabricated by the method of present study is 327.4 MPa. This is much higher than that fabricated by the molten resin method and the 3D printing method, which is 189.8 and 240.66 MPa, respectively [35,36]. It is also slightly higher than that produced by hot pressing method using woven prepreg tapes, which is 310 MPa [37]. The tensile strength of C-CFRTP is 546.2 MPa. This is approximately 3.7 and 3.1 times higher than that manufactured by 3D printing technology and injection molding, respectively, which used long fiber as reinforcing fiber, [21,38]. A higher value of 380 MPa was recorded using the film-stacking method [39]; however, this is still lower than that of the specimen fabricated by the method of the present study.

Figure 6 demonstrates the effect of the type of matrix resin on the flexural property of the C-FRP composite. The fabrication method and raw materials of specimens for flexural test are the same as that of the specimens for the tensile test which is mentioned above. Although the value of fiber volume fraction, which is shown at the bottom of Figure 6, is different from that for the tensile test, even when fabricated by same method and raw materials, the tendency is that the fiber volume fraction of C-FRTP is higher than that of C-FRP. Orange pillars show the flexural strength of C-GFRP, C-GFRTP, C-CFRP and C-CFRTP, while violet pillars show the flexural modulus of these specimens. C-GFRP and C-GFRTP, C-CFRP and C-CFRTP show similar flexural strength. The flexural modulus of C-GFRP is lower than that of C-GFRTP, and the flexural modulus of C-CFRP is a little lower than that of C-CFRTP. As with the result of tensile strength, this is also an exciting result, which was beyond expectation. As mentioned above, the fiber volume fraction, the mechanical properties of the reinforcing fibers and the matrix resin, and interfacial property between the reinforcing fiber and the matrix resin also influence the flexural property of the FRP composites. It can be observed from Figure 6 that the tendency of flexural property was almost the same as the tensile property. Although thew fiber volume fraction of C-GFRTP and C-CFRTP was higher, their flexural strength is similar to that of C-GFRP and C-CFRP. This is also mainly attributed to the low interfacial bond strength of C-GFRTP and C-CFRTP for the surface sizing agent was not suitable with polyester resin. The flexural modulus of C-GFRTP and C-CFRTP are also higher than C-GFRP and C-CFRP. However, this is not as obvious as the tensile modulus. This could be attributed to the bending experiment itself. As we know, the upper side of the specimen is subjected to compressive stress, and the lower side of the specimen is subjected to tensile stress in the bending test. Under compressive stress, the modulus of reinforcing fibers and resins are not significantly different, which lead to these results. The flexural strength of C-GFRTP fabricated by the method of present study is 321.5 MPa. This is higher than that manufactured by the molten resin method and the hot-pressing method using woven prepreg tapes, which is 193.7 and 288 MPa, respectively [35,37]. This is almost the same as that produced by the 3D printing method, which is 325.64 MPa [36]. The flexural strength of C-CFRTP is 603.2 MPa. This is approximately 4.7 and 1.6 times higher than that manufactured by 3D printing technology and the film-stacking method, respectively [21,39].

Although the tensile property and flexural property of the prepared C-FRTP are lower than that of C-FRP on the premise of the same fiber volume fraction, the tensile property and flexural property of the C-FRTP are nearly the same as that of C-FRP and have been significantly improved compared with previously reported C-FRTP with a low fiber volume fraction [40]. Hence, the current method is viable and enhances the mechanical property of C-FRTP effectively.

### 3.3. Impregnation Morphology

**Figure 7:**
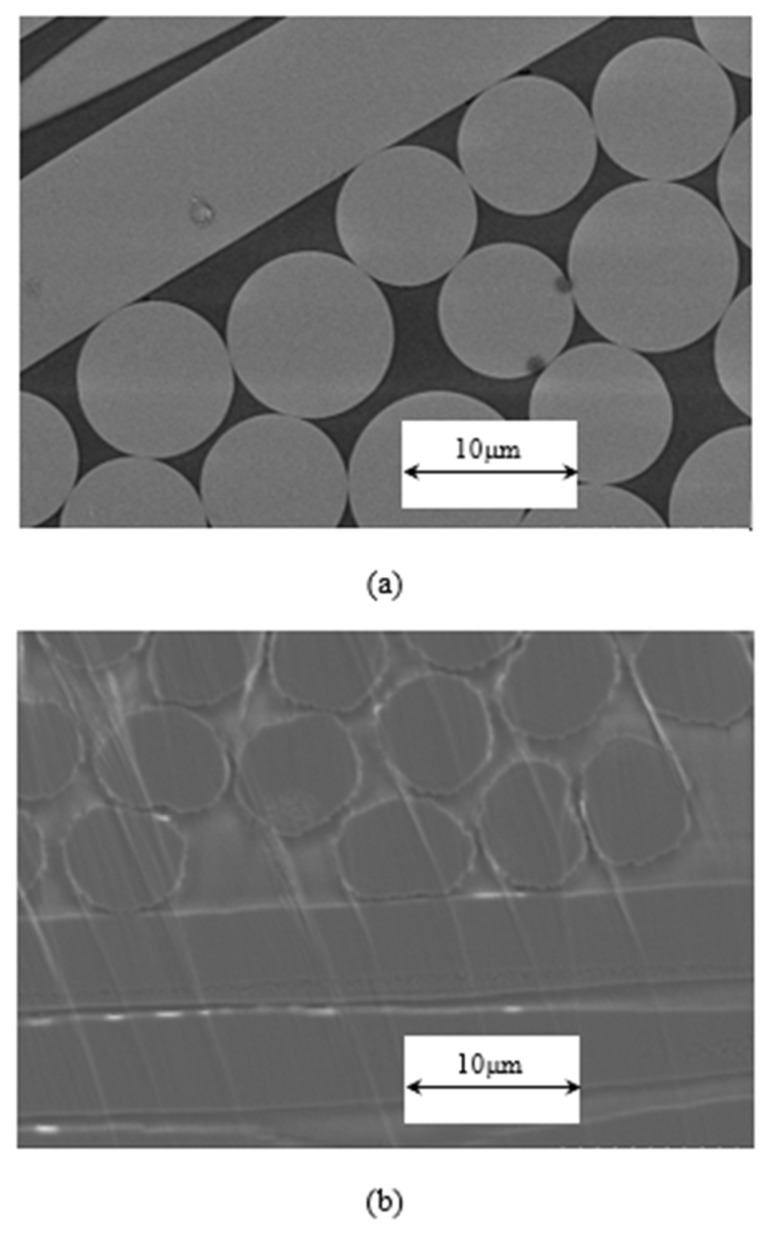
SEM images of cross section of (**a**) C-GFRTP and (**b**) C-CFRTP.

An ion-milling system was employed to precisely polish cross section of specimens. Additionally, the impregnation morphology of the thermoplastic polyester resin was determined by SEM observation. The SEM images are shown in Figure 7. The circular image is the fiber cross section, and the long image is the fiber longitudinal section. The polyester resin is between the fibers. It can be clearly observed that the fibers are filled with polyester resin, and there is no void between fibers caused by poor impregnation. Figure 7a is cross section of C-GFRTP, and a typical circular section can be seen. They are glass fiber with an average diameter of approximately 10 μm. Figure 7b is cross section of C-CFRTP, and a typical jagged section can be seen. This is caused by narrow grooved streaks that are parallel to the longitudinal direction of fibers. They are carbon fiber with an average diameter of approximately 7 μm. It can be seen from Figure 7 that the interface of both the C-GFRTP and C-CFRTP is tightly bound. There are no gaps between the fiber and the resin, which reveals that the load transfer well from resin to fiber. At the same time, there are no voids be found in resin. This plays a key role in reducing stress concentration. In view of the above, the low viscosity of the thermoplastic polyester resin resulted in good impregnation, which lead to improvement of the mechanical properties.

### 3.4. Fracture Surface Morphology Observation

**Figure 8:**
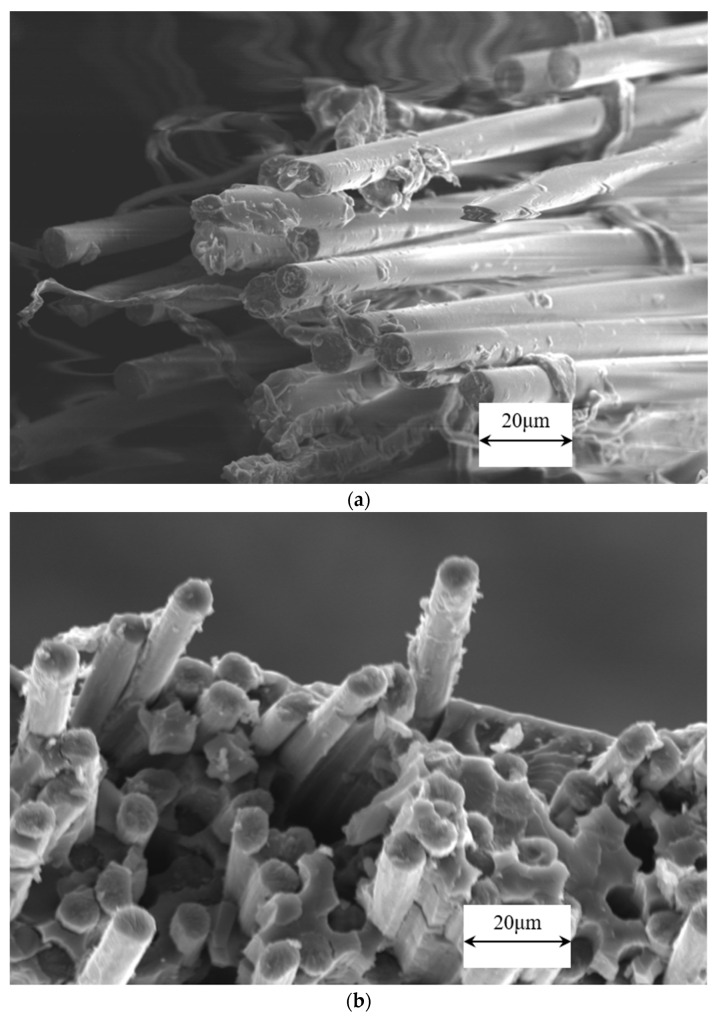
SEM images of fracture surface morphology of (**a**) C-GFRTP and (**b**) C-CFRTP.

Fracture surface morphology of the specimens was observed to determine the reason of composites fracture. Figure 8 show SEM images of fracture surface morphology after the mechanical test, (a) C-GFRTP, (b) C-CFRTP. Figure 8a shows a great deal of clean glass fibers without any polyester resin residue on them. Figure 8b shows many carbon fibers are removed from polyester resin. Additionally, there is also very little resin residue around them. This reveals that the fracture was mainly caused by a low level of interfacial bond between reinforcing fiber and polyester resin. This is mainly attributed to the fact that the surface sizing agent on reinforcing fiber was not suitable with polyester resin, which caused interfacial failure under loading. 

**Figure 9:**
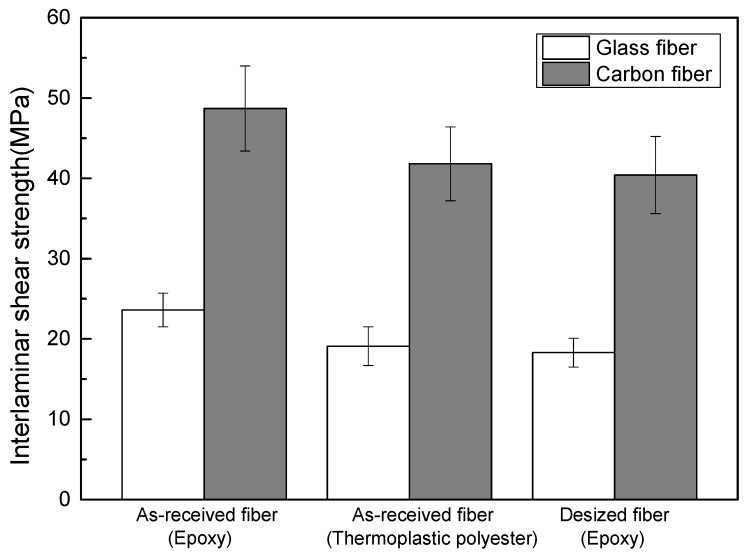
Average ILSS value of specimens.

The directly purchased reinforcing fiber was defined as as-received fiber, the surface treatment agent removed reinforcing fiber was defined as desized fiber. They were fabricated into composites reinforced epoxy resin and thermoplastic polyester resin, respectively. These composites were subjected to interlaminar shear strength (ILSS) evaluation. The results are shown in Figure 9. Whether it is glass fiber or carbon fiber, the ILSS of as-received fiber/epoxy resin is the highest. Additionally, the ILSS of as-received fiber/thermoplastic polyester resin is only slightly higher than that of desized fiber/epoxy resin, and is almost at the same level. That is, the surface treatment agent carried out by the manufacturer has little effect on the fiber/thermoplastic polyester resin. Obviously, surface treatment or a surface sizing agent that is suitable with resin is needed for practical applications in the future.

## 4. Conclusions

The above-mentioned results already allow us to conclude that it is possible to manufacture C-FRTP without void from almost all kinds of thermoplastic resin pre-impregnated materials which are prepared by the method developed by the present work. 

Currently, our work is carried out to decrease the viscosity of thermoplastic resin to values in the range from 1 to 5dPa×s. This will increase the liquidity of the thermoplastic resin to increase the fiber volume fraction and decrease the amount of void to enhance the mechanical property of the final C-FRTP composites. The fiber volume fraction was increased to approximately 60% by the present work compared approximately 40% that was fabricated by VaRTM method. From the results of mechanical evaluation, the tensile property and flexural property were enhanced to the same level with C-FRP.

The fracture surface morphology revealed that fibers are removed from polyester resin with a clean surface without any resin residue, indicating low level of interfacial bonding. Studies of the surface treatment or surface sizing agent that are suitable with resin are needed in order to increase the interfacial bonding property for future applications.

If the method proposed in this research is perfected, it will accelerate the application of lightweight composite materials in transportation fields, such as automobiles, aeroplanes, and watercrafts, and achieve the goal of reducing CO_2_ emissions, thereby achieving the aims of SDGs.

## Figures and Tables

**Table 1:** The physical properties of matrix resin.

Matrix Resin	Specific Gravity	Tg	Melting Point	Melt Viscosity
Thermoplastic polyester resin	1.28	78 °C	185 °C	7000 dPa×s/250 °C
Thermosetting epoxy resin	1.06	90 °C	—	3.48 dPa×s/25 °C

**Table 2:** The physical properties of reinforcing fiber.

Reinforcing Fiber	Density	Average Diameter	Area Density	Weave Density
Glass fiber	2.60 g/cm^3^	10 μm	200 g/m^2^	19 piece/25 mm (warp) 18 piece/25 mm (weft)
Carbon fiber	1.76 g/cm^3^	7 μm	198 g/m^2^	12.5 piece/25 mm (warp) 12.5 piece/25 mm (weft)

**Table 3:** Fiber volume fraction of all specimens.

FRP Composites	C-GFRP	C-GFRTP	C-CFRP	C-CFRTP
Fiber volume fraction (%) for tensile test	33.9	56.4	42.3	57.2
Fiber volume fraction (%) for flexural test	34.2	58.7	44.3	59.1

## Data Availability

No new data were created or analyzed in this study. Data sharing is not applicable to this article.
